# Students’ psychological biases towards teacher and AI-generated feedback: an experimental study

**DOI:** 10.1186/s40359-026-04568-5

**Published:** 2026-04-22

**Authors:** Haiyan Yu

**Affiliations:** Center for Linguistics and Applied Linguistics/Center for Lexicographical Studies, Guangdong University of Foreign Studies, No. 2 Baiyundadaobei, Guangzhou, 510420 China

**Keywords:** Subjective bias, Feedback source, AI-generated feedback, Teacher feedback, International students, Chinese as a second language

## Abstract

**Background:**

While AI-generated feedback has been promoted as a supplement to teacher feedback, recent empirical studies have consistently revealed that students prefer teacher feedback to AI-generated feedback. It remains unclear whether such preferences reflect the inherent quality of the feedback or stem from psychological biases toward the feedback source.

**Method:**

This study investigates the existence of psychological biases towards teacher and AI-generated feedback. An experiment was conducted with 52 advanced learners of Chinese as a second language (CSL). The subjects were randomly divided into two groups: AI-label group and teacher-label group. All participants received identical AI-generated feedback on an essay, but were led to believe it came from either an AI system (AI-label group) or an experienced teacher (teacher-label group). Data included participants’ perceptions of the feedback measured across seven psychological dimensions (coverage, accuracy, elaboration, utility, cost, interest, and intention), as well as their subsequent textual revisions coded by type and frequency.

**Results:**

Participants in the teacher-label group reported consistently more positive perceptions of the feedback across all dimensions, although these differences did not reach statistical significance. However, the AI-label group made significantly more textual revisions, particularly replacements, than the teacher-label group (*p* = .017). This reveals a dissociation between cognitive appraisal of feedback and behavioral engagement with it.

**Conclusion:**

These findings provide empirical evidence of a psychological bias against AI-generated feedback, wherein the same feedback is perceived less favorably when attributed to AI. However, this negative bias does not translate into reduced behavioral engagement; instead, learners interact more actively with AI-attributed feedback, potentially due to reduced social-evaluative concerns and enhanced autonomy. The study contributes to the psychology of human-AI interaction in educational contexts and highlights the need to consider both cognitive and socio-affective mechanisms in understanding feedback engagement.

**Supplementary Information:**

The online version contains supplementary material available at 10.1186/s40359-026-04568-5.

## Introduction

The rise of generative artificial intelligence (GenAI) has opened up promising new possibilities for enhancing second language (L2) writing pedagogy [30, 37, 56–58, 60]. AI-generated feedback is increasingly recognized for its potential to offer fast, consistent, and scalable support to L2 writers [43, 53, 55]. In response to these affordances, scholars have started examining whether AI-generated feedback can effectively supplement or even replace teacher feedback [54]. However, AI-generated feedback has often been viewed as less than satisfactory by its users [31]. Recent studies keep pointing in the same direction: students tend to value teacher feedback more deeply, and they engage with it more seriously. That’s not to say they don’t see AI’s strengths, such as accessibility, immediacy, comprehensiveness, but those advantages don’t seem to tip the balance [20, 60].

From a psychological perspective, this preference pattern raises a fundamental question: Do students’ differential responses to AI and teacher feedback reflect genuine differences in feedback quality, or do they stem from pre-existing psychological biases toward the feedback source? To address this question, the present study integrates three complementary theoretical frameworks that are already established in the feedback literature: source credibility theory [35], which elucidates how perceived expertise and trustworthiness shape message acceptance; social-evaluative threat [13, 14], which illuminates the anxiety students may experience when being evaluated by authority figures and their subsequent impression management strategies, and self-determination theory [59], which highlights how supporting learner autonomy fosters the intrinsic motivation necessary for proactive feedback engagement. Together, frameworks provide a systematic lens not only to detect the presence of source bias but also to unpack the underlying psychological mechanisms, particularly impression management and autonomy satisfaction, that may drive differential responses to AI and teacher feedback.

A key limitation in prior comparative studies is that the feedback content itself wasn’t really controlled for. It remains uncertain whether differences in students’ preferences and subsequent revisions are driven by the actual content of the feedback or by subjective perceptions of its source. This distinction matters. Feedback from automated systems, whether it’s something like Grammarly or a conversational tool like ChatGPT, has often been seen as focusing mostly on surface issues and lacking context [41, 46, 58]. Such negative perceptions may be amplified when AI systems are used for languages other than English, where available linguistic resources are comparatively limited [40]. For example, while ChatGPT has outperformed teachers in detecting errors and providing detailed, balanced feedback on English writing, it may underperform in other low-resource languages, where its comments were less balanced and occasionally inaccurate [11]. These results underscore the need to investigate the performance of AI-generated feedback and its perceptions beyond English. The suboptimal quality of AI-generated feedback may lead to persistent negative perceptions, to the extent that simply labeling feedback as AI-generated can bias students’ evaluations of feedback message [5, 38].

Despite recent interest in the topic of subjective bias in feedback perceptions, the few relevant studies have focused on perceived trustworthiness, with other dimensions of feedback perceptions underexplored. For instance, Lipnevich & Smith [28] demonstrated that students rated identical feedback as more accurate and helpful when attributed to a teacher rather than a computer, but their study did not examine the psychological mechanisms driving this bias or its behavioral consequences. Similarly, Ruwe & Kuklick [38] found that an AI label reduced perceived trustworthiness, yet they did not investigate how such bias might affect students’ actual revision behaviors or whether it extends to other perceptual dimensions such as interest, utility, or cost. The present study extends beyond this prior work by (a) examining bias across multiple cognitive and affective dimensions of feedback perception, (b) measuring behavioral engagement through textual revisions.

The present paper addresses this research gap by exploring whether a psychological bias exists in students’ perceptions of AI-generated feedback within the context of learning Chinese as a second language, and whether such bias influences their subsequent behavioral engagement with the feedback. Two groups of learners received identical feedback produced by an AI system but are informed of different feedback sources (AI vs. teacher). Specifically, this paper answers two questions:

1. Does the label of the feedback source influence students’ perceptions of the feedback?
2. Does the label of the feedback source influence students’ textual revisions in response to the feedback?

## Literature review

### The quality and functions of AI-generated feedback

Feedback has been identified as a key support for student learning [19]. However, in many low-resource educational contexts, quality teacher feedback is often unavailable or delayed. Automated writing evaluation (AWE) systems emerged to address this problem by providing scalable, consistent, and timely feedback to learners [2, 41]. Despite these affordances, AWE feedback has often been viewed as inferior to teacher feedback. Research on established AWE tools such as *Pigai*, *Grammarly*, and *Criterion* reveals persistent limitations in their ability to identify and explain errors accurately [9]. For instance, Bai and Hu [3] found that *Pigai* produced correct suggestions in only 58.61% of grammar cases and 21.83% of collocation cases, confirming AWE’s tendency to emphasize surface-level issues over higher-order concerns such as idea, coherence, and style [46, 60].

Recently, GenAI marks a turning point in AWE feedback. Tools such as ChatGPT are believed to move beyond rule-based error detection to emulate human-like evaluation of ideas, argumentation, and discourse structure [32]. Comparative studies, however, show mixed results about the quality of GenAI-generated feedback [6]. Steiss et al. [43] compared feedback from human evaluators and ChatGPT 3.5 on a corpus of English essays and found that human feedback scored higher on most quality dimensions, including accuracy, prioritization, and supportive tone. They noted that ChatGPT, though more consistent in referencing assessment criteria, occasionally produced inaccurate or contradictory comments. Similarly, Fokides and Peristeraki [11] observed that while ChatGPT surpassed teachers in error detection for English essays, it underperformed in Greek writing due to inaccurate flagging and less attention to mechanics. These findings suggest that GenAI’s capacity to generate high-quality feedback varies across languages, especially those with limited training resources.

Overall, this body of research reveals both the potential and the limitations of AI-mediated feedback. While AI can provide high-volume, rapid responses, it still faces challenges with linguistic nuance, contextual understanding, and cross-linguistic equity.

### Potential and uptake of AI-generated feedback

AWE has the potential to promote L2 writing. AWE systems can promote noticing, provide metalinguistic explanations, and encourage self-directed learning [4, 24]. Empirical evidence shows that learners who receive AWE feedback often outperform those who do not, particularly in grammatical accuracy (e.g., [4]). Yet, findings comparing AWE and teacher feedback remain inconclusive. Some studies report stronger effects of AWE on writing quality (e.g., [18]), while others find no significant difference (e.g., [10]) or highlight complementary effects when AWE is combined with teacher input [17, 58]. These results suggest that AWE is most useful as a *collaborative tool* rather than a replacement for teachers [20].

However, feedback is not useful until it is properly utilized [52]. While AI can provide timely and scalable feedback, there is no guarantee that students can make good use of the feedback provided, especially given the large amount of feedback. Feedback can be easily interpreted as criticism even if the feedback focuses on the areas for improvement. Excessive critical feedback can induce anxiety and impede feedback uptake [26, 44]. Studies consistently show low uptake rates of AI-generated feedback: learners adopt only about half of the system’s suggestions [3, 47]. For example, Koltovskaia [25] found that their participants corrected about 57% of the errors flagged by *Grammarly*. Low uptake has been attributed not only to technical limitations, such as inaccuracy and lack of metalinguistic explanation [4, 15], but also to learners’ *perceptions* of the credibility and usefulness of AWE feedback [27]. For instance, Zou et al. [60] found that students incorporated more revisions based on teacher feedback than on AI-generated feedback. These findings highlight that feedback uptake is not only a cognitive process but also a socially and affectively mediated one, dependent on learners’ trust and perceived value of the feedback source [36].

### Feedback source, psychological biases, and feedback perceptions

Research has shown that the source of feedback can impact students’ perceptions of the feedback they receive [50]. This influence may arise from the characteristics of the feedback itself or from the credibility of its source (van [49]). However, beyond these direct effects, students’ responses to feedback may also be shaped by psychological biases: systematic tendencies in information processing that deviate from rational judgment, often influenced by heuristics, social stereotypes, or prior beliefs [23].

Two theoretical perspectives help explain why such biases might operate in feedback contexts. First, source credibility theory suggests that the perceived expertise, trustworthiness, and goodwill of the feedback provider significantly influence how feedback is received [35]. Students may automatically attribute higher credibility to human teachers based on their social status and institutional authority, while AI systems, despite their technical capabilities, may be viewed as lacking authentic understanding of learners’ needs. Second, social identity theory [45] offers another perspective: teacher feedback is embedded within an existing social relationship where students’ identities as learners are recognized and affirmed, whereas AI-generated feedback, being impersonal, may fail to engage students’ social identity, leading to differential processing of the same information.

In the case of AI-generated feedback, both students and teachers have reported mixed perceptions regarding its characteristics [12]. On one hand, some students perceive AI-generated feedback as more timely and detailed than teacher feedback [33]. AI-generated feedback helps students address surface-level issues such as typos and grammatical errors while providing guidance for improvement and textual revisions [1]. On the other hand, students often perceive AWE feedback as inferior to teacher feedback because it may lack accuracy and explicitness and has limited capacity to offer in-depth feedback on content, coherence, style, and overall precision [12, 44, 51].

Notably, studies have demonstrated that such perceptions may reflect biases rather than genuine quality differences. Lipnevich & Smith [28] found that students rated teacher feedback as significantly more accurate and helpful than computer-generated feedback, even though the feedback was identical in content. This classic finding points to the operation of psychological bias: the same message is evaluated differently based solely on its attributed source.

The mixed perceptions associated with traditional AI-generated feedback systems also apply to AI. Recent research has examined how students perceive feedback generated by AI in comparison to teacher feedback. For example, Escalante et al. [10] examined the use of ChatGPT-4 among 48 tertiary EFL learners over six weeks. Their study suggested that students generally preferred teacher feedback over AI-generated feedback. Similarly, Zou et al. [60] investigated how Chinese EFL students responded to feedback on their English writing when it was provided by teachers versus generated by ChatGPT. Students perceived teacher feedback as more helpful than AI-generated feedback; however, only the difference in perceived usefulness of feedback for language use was statistically significant. Overall, teacher feedback was preferred over AI-generated feedback. In contrast, Henderson et al. [20] administered a survey of 6,960 students from four Australian universities. They found that half of the students sought feedback from GenAI, and that students perceived teacher feedback to be more useful and more trustworthy.

Recent experimental evidence has further isolated the role of source attribution. Ruwe and Kuklick [38] showed that participants rated feedback labeled as AI-generated as less trustworthy than feedback labeled as teacher-generated, without knowing the source of feedback. Similarly, Brummernhenrich et al. [5] found that simply labeling feedback as AI-generated reduced students’ perceptions of its trustworthiness, regardless of its actual quality. These findings suggest that psychological biases toward feedback sources persist even as AI technologies become more sophisticated. Students might assume that AI-generated feedback is less accurate than teacher feedback and develop a sense of distrust toward it [12, 44].

Such stereotypes not only influence students’ judgements regarding the authority and relevance of AI-generated feedback but also potentially influence students’ response to AI-generated feedback. User perceptions of feedback can play a key role in feedback utilization [52]. Students evaluate the quality of feedback and weigh the costs and benefits of seeking and implementing it [13]. Their subjective perceptions of AWE feedback influence their subsequent feedback uptake. Although artificial intelligence has become more powerful than ever, and AI-generated feedback appears comparable to human-generated feedback [11], language learners may still harbor lingering biases regarding AWE feedback and its functions [12, 44].

While the potential biased view of AI-generated feedback has far-reaching implications, limited research has been conducted to systematically isolate the effect of source attribution while controlling for feedback content. Moreover, most bias research has focused on trustworthiness perceptions, leaving other dimensions of feedback perception (e.g., perceived utility, interest, cost) underexplored. To address this gap, the present paper reports on an experiment that aims to determine whether CSL learners hold biased perceptions of AI-generated feedback and whether the designated source of the feedback affects their revisions.

## Methodology

### Participants and the context

A total of 52 CSL learners were invited to participate in the experiment. All participants were international students studying Chinese at a public university in China. They came from multiple countries: Indonesia (24), Myanmar (4), Thailand (4), Venezuela (3), the Dominican Republic (2), Mongolia (2), Peru (2), Japan (2), Vietnam (2), Belarus (1), Russia (1), France (1), Kyrgyzstan (1), Canada (1), Malaysia (1), and South Africa (1). Seventy-five percent of the participants (39) are descendants of overseas Chinese. All participants demonstrated advanced Chinese proficiency, as evidenced by a score of ≥ 220 on the HSK, an international standardized test for Chinese language proficiency. They had normal or corrected-to-normal visual acuity. They were informed of the experimental procedures and gave written consent to participate. But they were naive to the purpose of the experiment, i.e., to test for subjective bias in feedback perceptions. Each participant received a small gift as a token of appreciation upon completing the experiment.

The sample size was determined based on guidelines proposed by Cohen [7], which recommends a minimum statistical power of 0.8 and a corresponding effect size for robust results. Using G*Power software, we estimated that a total sample size of 50 participants was required for a one-factor, two-level between-subjects design. To account for potential attrition, we recruited 52 participants, comprising 18 males and 34 females, with an average age of 21.90 years (*SD* = 2.22). Participants were randomly allocated to two groups. The first group (hereafter Group 1) was informed that the feedback they processed was generated by an AI system. The second group (hereafter Group 2) was told that the feedback they processed was composed by an experienced teacher.

To ensure that the random assignment successfully balanced participants’ Chinese proficiency across the two experimental groups, an independent samples *t*-test was conducted on their HSK scores. The results revealed no significant difference between the AI-label group (*M* = 254.31, *SD* = 18.65) and the teacher-label group (*M* = 247.88, *SD* = 19.38), *t*(50) = −1.22, *p* = 0.23, 95% CI [− 17.02, 4.17], Cohen’s *d* = 0.34. This indicates that the two groups were comparable in terms of Chinese language proficiency prior to the experiment, thereby ruling out proficiency as a potential confounding variable in subsequent analyses.

### Materials

The materials include an essay and the feedback comments that an AI system generated for the essay. The essay was selected through a systematic procedure. First, three essays, representative of high, medium, and low proficiency levels, were randomly selected from the Dynamic Composition Corpus for HSK, developed by Beijing Language and Culture University. The three essays were evaluated by five senior CSL instructors, each with at least seven years of teaching experience, to assess their suitability for the experiment. The essay titled *The Impact of Smoking on Personal Health and Public Welfare* was chosen because it was typical of the proficiency level of the participating students. The selected essay was presented to two advanced Chinese learners and an experienced CSL teacher, who were invited to revise it. Their textual changes were used to ensure that the essay requires revisions.

Prior to the expert review, selection criteria and evaluation standards were established. Three criteria guided the initial screening: (1) the essay should be representative of the target proficiency level; (2) it should contain identifiable areas for improvement in terms of content, organization, and language use; and (3) its length should be appropriate (approximately 350 characters) for completion within the experimental timeframe.

The essay was then submitted to Kimi AI Chat, a free Chinese AI platform, which was instructed to assess the essay and provide feedback based on the HSK rubric. The prompt was “Please provide feedback for the essay based on the HSK rubric attached below”. The feedback points provided by Kimi AI Chat can be found in Appendix B. The feedback was used in its original form without any editing or modification. This decision was made deliberately to preserve ecological validity, that is, to reflect the kind of feedback students would actually receive when interacting with an AI system in authentic learning contexts. The feedback consisted of seven evaluative points and five revision suggestions (see Appendix B), which covered both global (content, structure, coherence) and local (grammar, vocabulary, mechanics) aspects of writing.

It is worth noting that the feedback was generated through a rigorous, multi-stage protocol using Kimi AI Chat. The procedure comprised three rounds:1) corpus preprocessing, text analysis, preliminary AI analysis, and expert evaluation against HSK criteria; 2) AI scoring, manual text optimization based on academic theories; 3) final AI scoring for quality verification. Throughout this process, the AI tool was strictly regulated for multi-dimensional analysis.

### Feedback perception questionnaire

Students’ feedback perceptions were measured using a composite questionnaire consisting of seven dimensions: coverage (5 items), accuracy (3 items), elaboration (4 items), interest (4 items), utility (3 items), cost (4 items), and intention (3 items). The items were adapted from validated instruments used in prior feedback studies ([13, 34, 50]) and revised to suit the context of this experiment. Responses were recorded on a 5-point Likert scale ranging from 1 (strongly disagree) to 5 (strongly agree).

The questionnaire demonstrated excellent internal consistency, with an overall Cronbach’s alpha of 0.899. All subscales exceeded the recommended reliability threshold of 0.80, indicating satisfactory measurement quality: coverage (α = 0.813), accuracy (α = 0.825), elaboration (α = 0.832), interest (α = 0.823), utility (α = 0.877), cost (α = 0.832), and intention (α = 0.824).

### Data collection and analysis

**Fig. 1:**
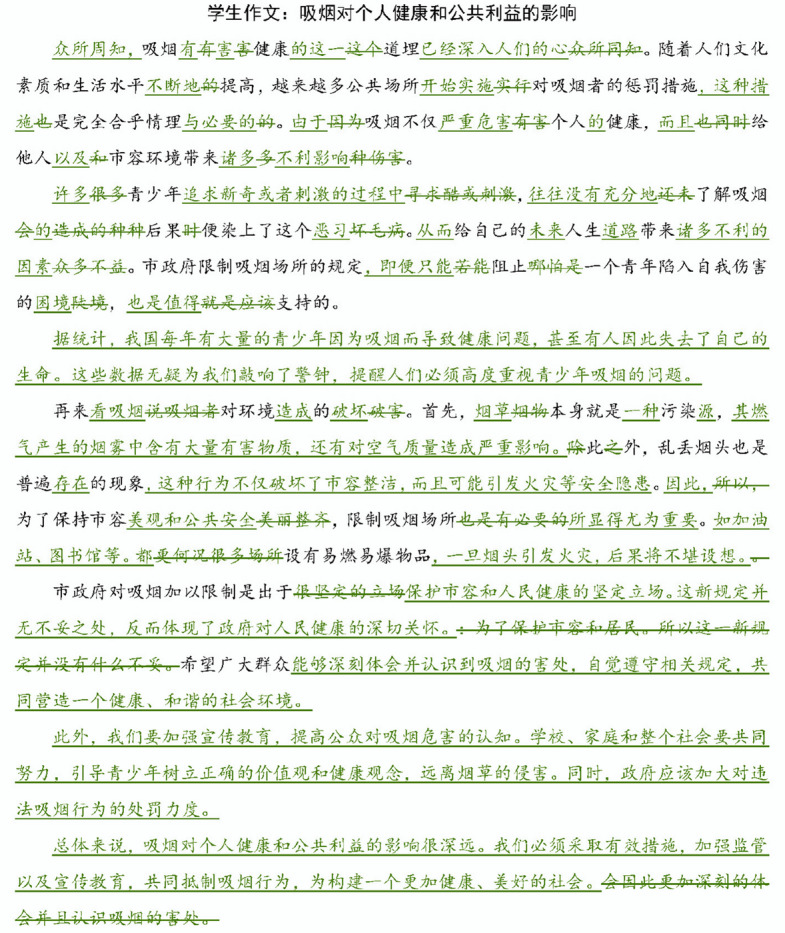
Revisions made by a student receiving feedback with the AI label

**Fig. 2:**
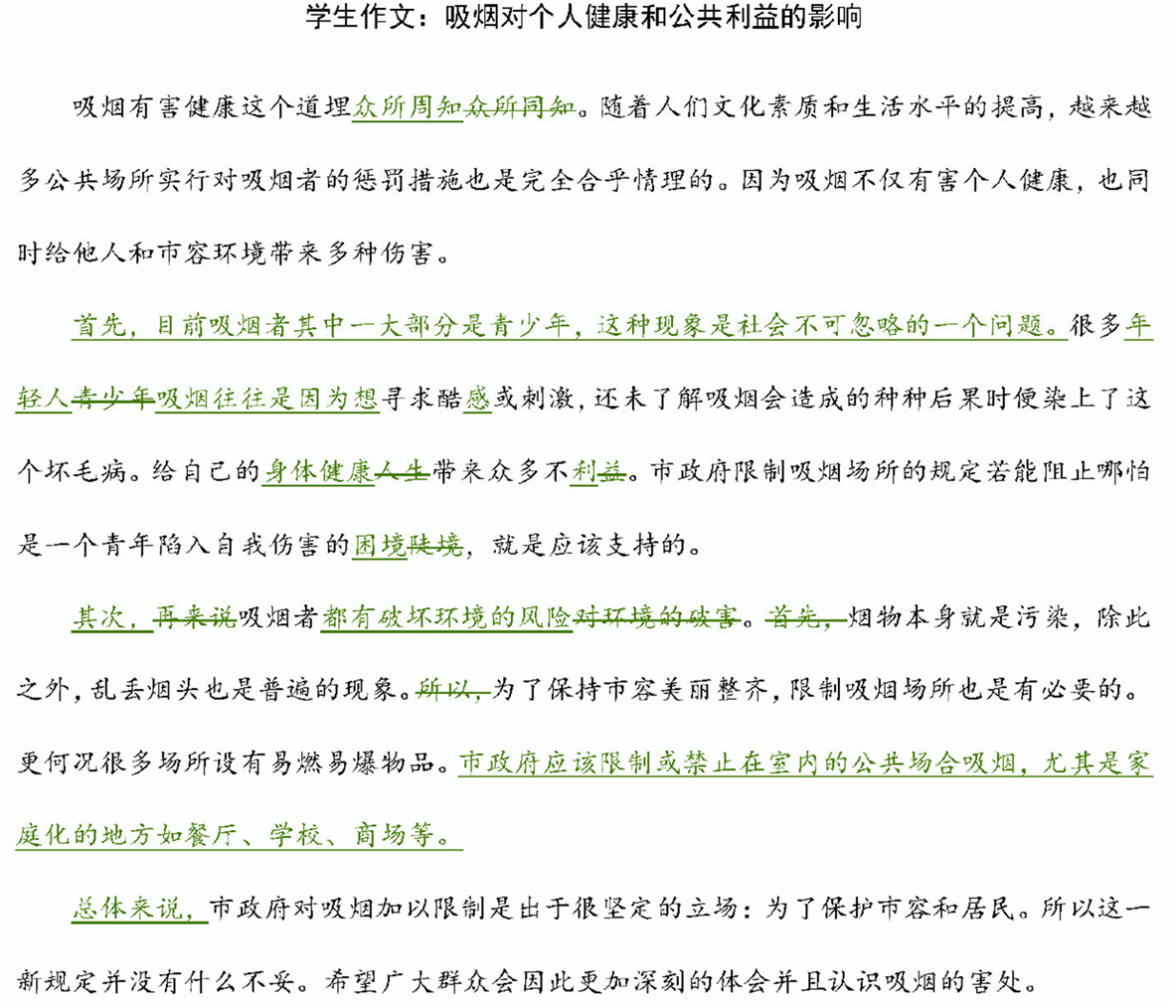
Revisions made by a student receiving feedback with the label of teacher feedback

The experiment was administered in a computer lab. Participants were first presented with the selected essay and the accompanying AI-generated feedback. Afterwards, they were asked to answer a questionnaire regarding their perceptions of the feedback. Fifteen minutes were given to read the essay and its accompanying feedback. The questionnaire took approximately five minutes to complete. Afterward, participants were asked to revise the essay based on the feedback they had received. The revision was completed using MS Word with the Track Changes feature enabled (see Figs. 1 and 2). All participants completed the revision within forty-five minutes.

The students’ revised essays were imported into MaxQDA, a qualitative analysis software used for coding. Textual changes were identified and coded based on meaning rather than on a clause-by-clause basis. A unit of analysis could be as short as a single Chinese character or as long as a full sentence. Once all units of analysis were identified, they were coded according to the type of revision: replacement, deletion, addition, or reordering. One author coded all the units of analysis twice, with a two-month interval between sessions. Intra-coder reliability was assessed to ensure consistency in coding. Another author reviewed each coding, and any disagreements were resolved through iterative discussions.Inter-rater reliability was evaluated using the intraclass correlation coefficient (ICC) with a two-way random effects model, absolute agreement, and single measurement. The ICC values for the four modification types were 0.97 (95% CI [0.956, 0.983]) for Replacement, 0.94 (95% CI [0.896, 0.964]) for Deletion, 0.99 (95% CI [0.984, 0.994]) for Addition, and 0.78 (95% CI [0.655, 0.863]) for Re-ordering. These values indicate excellent to good reliability, and all were statistically significant.

## Results

The normality of the data was first assessed. No severe violation of the normality assumption was detected. An independent samples *t*-test was performed to compare the feedback perceptions between Group 1 (AI-label) and Group 2 (teacher-label) across several measures. Descriptive statistics, inferential test results, effect sizes (Cohen’s* d*), and 95% confidence intervals are presented in Table 1.

**Table 1:** Comparison of feedback perceptions

Measure	Group 1 M (*SD*)	Group 2 M (*SD*)	*p*	Cohen’s *d*	95% CI
Coverage	4.12 (0.58)	4.18 (0.66)	.716	0.10	[−0.415, 0.287]
Accuracy	3.84 (0.75)	4.17 (0.69)	.107	0.46	[−0.741, 0.075]
Elaboration	3.93 (0.73)	3.96 (0.78)	.889	0.04	[−0.399, 0.459]
Utility	4.29 (0.74)	4.39 (0.63)	.633	0.15	[−0.484, 0.297]
Cost	2.51 (1.00)	2.41 (1.04)	.747	−0.10	[−0.485, 0.672]
Interest	3.54 (0.96)	3.82 (0.82)	.460	0.31	[−0.785, 0.225]
Intention	4.44 (0.70)	4.28 (0.82)	.271	−0.21	[−0.272, 0.592]

Group 1: AI group; Group 2: Teacher group. Cohen’s *d* was calculated using pooled standard deviation. Positive *d* values indicate higher mean scores for the teacher-label group; negative *d* values indicate higher mean scores for the AI-label group. Effect sizes are interpreted as small (0.2), medium (0.5), and large (0.8) according to Cohen (1988)

As shown in Table 1, no statistically significant differences were found between the two groups on any of the measures. However, examination of mean scores and effect sizes revealed patterns consistent with potential bias toward the feedback source. For perceived accuracy, a small-to-medium effect was observed favoring the teacher-label group (*M* = 4.17, *SD* = 0.69) over the AI-label group (*M* = 3.84, *SD* = 0.75), Cohen’s *d* = 0.46, 95% CI [−0.74, 0.08]. Small effects favoring the teacher-label group were also found for coverage (Cohen’s *d* = 0.10), elaboration (Cohen’s *d* = 0.04), utility (Cohen’s* d* = 0.15), and interest (Cohen’s *d* = 0.31). Additionally, feedback was perceived as slightly less costly when attributed to a teacher (Cohen’s d = −0.10).

Remarkably, the only dimension on which the AI-label group reported a higher mean was intention to receive additional feedback (*M* = 4.44, *SD* = 0.70) compared to the teacher-label group (*M* = 4.28, *SD* = 0.82), yielding a small effect in the opposite direction (Cohen’s *d* = −0.21, 95% CI [−0.27, 0.59]). While none of these differences reached statistical significance, the pattern of effect sizes, particularly for accuracy (Cohen’s *d* = 0.46) and interest (Cohen’s *d* = 0.31), suggests small to medium practical effects that are psychologically meaningful.

Turning to the analysis of textual revisions, an independent samples *t*-test was used to compare the revisions made by Group 1 (AI group) and Group 2 (Teacher group) across several measures (see Table 2). Descriptive statistics, significance tests, effect sizes (Cohen’s *d*), and 95% confidence intervals are presented in Table 2.

**Table 2:** Comparison of revisions

Measure	Group 1 M (*SD*)	Group 2 M (*SD*)	*p*	Cohen’s *d*	95% CI
Replacement	11.00 (6.35)	7.62 (2.53)	.017	−0.70	[0.656, 6.113]
Deletion	2.42 (2.28)	1.69 (1.98)	.223	−0.34	[−0.459, 1.920]
Addition	8.88 (7.10)	7.12 (4.74)	.296	−0.29	[−1.595, 5.133]
Re-ordering	0.69 (0.65)	0.79 (1.13)	.887	0.11	[−0.504, 0.581]

Same as Table 1

As shown in Table 2, a statistically significant difference was found between the two groups for replacements, *t* (50) = 2.48, *p* = 0.017, with the AI-label group making significantly more replacements (*M* = 11.00, *SD* = 6.35) than the teacher-label group (*M* = 7.62, *SD* = 2.53). The 95% confidence interval for the mean difference [0.66, 6.11] did not cross zero, further confirming the reliability of this effect. The effect size was medium to large (Cohen’s *d* = −0.70), indicating that this difference is not only statistically significant but also practically meaningful.

For the remaining revision types, no statistically significant differences were observed. However, the pattern of means and effect sizes revealed consistent trends favoring the AI-label group. Specifically, the AI-label group made more deletions (*M* = 2.42, *SD* = 2.28) than the teacher-label group (*M* = 1.69, *SD* = 1.98), *t*(50) = 1.24, *p* = 0.223, with a small effect size (Cohen’s* d* = −0.34, 95% CI [−0.89, 0.20]). Similarly, the AI-label group made more additions (*M* = 8.88, *SD* = 7.10) compared to the teacher-label group (*M* = 7.12, *SD* = 4.74), *t*(50) = 1.06, *p* = 0.296, also yielding a small effect (Cohen’s d = −0.29, 95% CI [−0.84, 0.25]). For re-ordering, the difference was negligible and slightly favored the teacher-label group (*M* = 0.79, *SD* = 1.13) over the AI-label group (*M* = 0.69, *SD* = 0.65), *t*(50) = −0.38, *p* = 0.887, with a minimal effect size (Cohen’s d = 0.11, 95% CI [−0.44, 0.65]).

Overall, students in the AI-label group implemented a larger total number of revisions than those in the teacher-label group, particularly in the category of replacements. This pattern reveals a dissociation between cognitive appraisal and behavioral engagement: while students perceived identical feedback less favorably when attributed to AI (see Table 1), they engaged more actively with it in terms of textual revisions.

## Discussion

This experimental study investigated the presence of bias in the perception and use of AI-generated feedback among CSL learners. Although most perceptual differences did not reach statistical significance, the pattern of effect sizes, particularly for accuracy (Cohen’s *d* = 0.46) and interest (Cohen’s *d* = 0.31), suggests small to medium practical effects that are psychologically meaningful. The findings reveal a psychologically significant dissociation between cognitive appraisal and behavioral engagement: while students perceived identical feedback more favorably when attributed to a teacher, they made significantly more revisions when the same feedback was labeled as AI-generated.

Consistent with previous research, students reported less positive perception of feedback across all dimensions when they were informed that it came from an AI system. This result aligns with a study by Lipnevich and Smith [28], who found that students perceived teacher feedback as more accurate and helpful than computer-generated feedback, even though the feedback was identical in content. To eliminate the possibility that participants’ judgments were biased by skepticism regarding the feedback source, participants who received feedback labeled as “teacher” were interviewed after experiment. The results indicated that they did not doubt the authenticity of the feedback source. Thus, such subjective bias appears to persist even today, notwithstanding that contemporary AI systems substantially outperform traditional automated writing evaluation (AWE) systems [20].

From a psycholinguistic standpoint, the persistence of this bias can be attributed to several interrelated factors. First, source credibility heuristics: cognitive shortcuts that lead learners to automatically assign higher authority to human sources based on social status and institutional roles [35], continue to operate even when AI demonstrates comparable or superior performance. Second, algorithm aversion [8] suggests that people are more tolerant of human errors than algorithmic errors; humans’ mistakes are often viewed as learning opportunities, whereas algorithmic errors tend to be perceived as fundamentally flawed. Third, social presence theory [42] posits that feedback from a perceived human source carries greater social and emotional weight, thereby activating deeper cognitive processing. Finally, cultural factors may amplify these biases: in Confucian-heritage contexts such as China, teachers are traditionally regarded as moral and intellectual authorities [21], which may heighten psychological resistance to accepting feedback from non-human evaluators. Together, these psycholinguistic mechanisms help explain why the bias against AI-generated feedback remains resilient despite technological advancements.

This study also aligns with Ruwe & Kuklick [38], who found that labeling feedback as AI-generated reduced its perceived trustworthiness. However,the present study extends this line of inquiry by demonstrating that an AI label diminishes positive perceptions across both affective and cognitive dimensions, ranging from interest to perceived accuracy, elaboration, and utility. This multidimensional impact warrants further elaboration. Perceived accuracy was affected because students may doubt AI’s capacity to understand nuanced language use, particularly in a second language context where pragmatic and cultural knowledge is required [11]. Perceived elaboration: the extent to which feedback explains errors and provides guidance, was rated lower for AI-generated feedback possibly because students assumed that machine-generated comments follow rigid templates and lack the contextual sensitivity of human feedback [6]. Perceived utility was influenced by the belief that teacher feedback better addresses individual learning needs and long-term writing development [60]. On the affective dimension, interest value was diminished for AI-generated feedback, likely because students perceive interaction with machines as less socially engaging and emotionally satisfying than interaction with teachers [5]. These findings demonstrate that source bias operates not as a monolithic construct but as a constellation of cognitive and affective judgments that collectively shape students’ overall evaluation of feedback quality.

Beyond confirming the existence of bias, this study advances research on student preferences for teacher feedback [20]. Specifically, prior research comparing perceptions of AI-generated feedback with those of teacher feedback has attributed differences in perceptions such as trust to the distinct characteristics of feedback from these sources (e.g., [20, 36, 48, 57]). The present study, however, shows that with a different label, students could have different perceptions of the same feedback. Overall, the differing perceptions caused by a labeled source point to the existence of subjective bias toward feedback sources.

Interestingly, the more positive perceptions elicited by the teacher feedback label did not lead to a higher level of engagement with the feedback. This finding challenges the intuitive assumption that positive cognitive appraisal directly translates into greater behavioral engagement, a phenomenon with important psychological implications. It contradicts assumptions that positive feedback perceptions regarding trustworthiness and feedback quality are associated with greater feedback engagement [20, 38]. Instead, this study demonstrated that students responded more actively to feedback when informed that it was provided by an AI system, despite expressing less favorable perceptions of the feedback. Specifically, the AI group enacted a larger number of revisions than the teacher group, with the difference in replacements reaching statistical significance.

Contrary to earlier findings (e.g., [29, 60]), which indicated stronger learner engagement with teacher feedback, this study revealed that AI-generated responses can elicit greater learner interaction in certain contexts. The discrepancy between more positive feedback perceptions and a lower level of feedback engagement suggests alternative explanations other than those proposed in prior research. For instance, Liu et al. [29] found that secondary school students who received AWE feedback on their Chinese writing made fewer revisions compared to peers who received feedback from teachers. The relatively fewer revisions when interacting with AWE feedback could be attributed to the overload caused by feedback quantity and its focus on surface issues. Zou et al. [60] found that students showed a higher uptake of teacher feedback on their English writing compared to feedback generated by ChatGPT. They attributed this higher rate of uptake to greater trust in the accuracy of teacher feedback. These explanations do not apply to the present study, in which both groups acted on the same feedback, yet students rated the feedback more positively when they were told it was from a teacher. In other words, trust in teacher feedback is not the sole reason for the higher level of engagement with teacher feedback. Alternative explanations are needed, and this study points to several specific mechanisms grounded in socio-affective and motivational psychology.

Among the four revision types, replacements emerged as the most sensitive indicator of engagement, yielding both statistical significance and a medium-to-large effect size (Cohen’s *d* = 0.70). This may be because replacements require deeper cognitive processing than deletions or additions: they involve not only identifying an error but also generating an appropriate alternative, which demands greater linguistic judgment and decision-making. Additions and deletions, while also indicative of engagement, can sometimes reflect more mechanical corrections (e.g., inserting a missing particle or removing a redundant character). The consistent pattern favoring the AI group across all three revision types, reinforces the overall finding of heightened behavioral engagement with AI-attributed feedback, even though only replacements reached statistical significance.

First, social-evaluative concern plays a crucial role. When feedback is attributed to a teacher, students become acutely aware of being evaluated by an authority figure, which activates impression management goals [13]. This awareness may paradoxically inhibit engagement, as students restrict their revisions to only those issues explicitly flagged by the teacher to avoid appearing presumptuous or risking the exposure of additional weaknesses. In contrast, AI-generated feedback, being free from social evaluation, reduces psychological costs and allows students to revise more freely.

Second, autonomy satisfaction, a key component of self-determination theory [39], may be higher with AI-generated feedback. Students interacting with AI perceive greater control over the revision process, as they are not constrained by the need to align with teacher expectations or manage interpersonal dynamics. This sense of autonomy fosters intrinsic motivation to engage with the feedback more extensively [59].

Third, self-presentation costs differ between the two sources. Revising in response to teacher feedback carries the risk of revealing one's limitations to someone whose opinion matters for academic success [14]. Students may therefore adopt a cautious approach to minimize potential face threats. With AI-generated feedback, this social cost is eliminated, enabling more experimental and expansive revision behaviors.

Fourth, achievement goal orientation may mediate the relationship. Teacher feedback tends to activate performance-avoidance goals (fear of appearing incompetent), whereas AI-generated feedback may promote mastery goals [34]. This differential goal activation explains why students engage more deeply with AI-generated feedback despite perceiving it as less credible.

Taken together, these alternative explanations collectively account for the dissociation between cognitive appraisal and behavioral engagement observed in this study, highlighting the need to consider socio-affective and motivational factors in understanding feedback uptake.

The findings also resonate with the broader socio-affective psychological framework [20, 38]. Teacher feedback involves teacher-student relationships [13, 16]. From a social psychology standpoint, the authority inherent in teacher feedback activates different motivational and emotional responses compared to AI-generated feedback. On the one hand, the authority of teacher feedback could decrease the likelihood of uninvited revisions, that is, revisions that are neither requested nor suggested in teacher feedback. In this experiment, most of the participants were descendants of overseas Chinese nurtured in Chinese culture. They tend to hold high regard for the authority of their teachers. This cultural orientation amplifies the psychological salience of authority and social evaluation. For them, the primary goal of revision behavior is to meet the teacher’s requirements and avoid negative evaluations. Due to their goal-oriented and risk-averse nature, they made only minimal corrections to the issues explicitly identified by the teacher. The assumption is that the absence of negative feedback indicates no serious issues with the writing. Unsolicited revisions may imply that teachers are neglecting their responsibilities and have overlooked important points. The threat to teachers’ authority is particularly alarming when unsolicited revisions can considerably enhance the quality of the writing. Thus, students tend to suppress “unnecessary” revisions to avoid causing dissonance between teacher feedback and the number of revisions. This behavior reflects impression management concerns and social-evaluative threat, well-documented psychological phenomena in feedback contexts. On the other hand, responding to teacher feedback may expose students’ shortcomings and thus incur self-presentation costs [13]. The embarrassment is particularly pronounced when unsolicited revisions fail to improve the writing or, even worse, degrade its quality. Such instances can reveal their deficiencies. Consequently, students were more cautious when revising text in response to teacher feedback than to AI-generated feedback. For segments without teacher feedback, participants assumed the text had no serious issues and therefore made fewer revisions, possibly to avoid unnecessary changes and protect their self-esteem.

In contrast, the socio-affective psychological factors are less salient in interactions with AI-generated feedback. There is minimal risk of revealing personal deficiencies to AI systems [20]. From a self-determination theory perspective, AI-generated feedback may better satisfy learners’ basic psychological needs for autonomy and competence**.** AI-generated feedback is generally perceived as inferior to teacher feedback. When interacting with AI-generated feedback, students are confident that they can outperform it. Consequently, they make more attempts to revise their texts. Due to its impersonal nature and lack of direct association with external rewards, punishments, or interpersonal evaluations, AI-generated feedback can satisfy learners’ autonomy needs, thereby fostering autonomous motivation [59]. Under this motivation, students revise their essays in response to AI-generated feedback with an intrinsic desire to prove their own abilities. Students demonstrate their language skills as superior to the AI tool through text optimization, achieving self-affirmation of human agency. It is no wonder that students addressing computer-generated feedback often display a higher level of self-confidence and a lower level of anxiety [29, 58]. This psychological state, characterized by reduced evaluative threat and enhanced intrinsic motivation, explains why revision behaviors extend beyond the scope of the feedback, exhibiting both high frequency and a broad scope when learners believe they are interacting with AI.

## Implications

This study offers important implications for research on AI-generated feedback. First, the finding that students perceived identical feedback less favorably when attributed to AI, suggests that future comparisons between AI and teacher feedback must account for potential subjective biases. It is crucial to differentiate between variations in learners’ engagement and subsequent learning outcomes that arise from biased perceptions of the feedback source from those that stem from the intrinsic qualities of the feedback itself. The bias is not constrained to trustworthiness or source credibility; it also relates to other aspects of feedback perception, such as perceived utility and cost [13, 14]. Such differentiation will enable a more precise understanding of how students perceive and enact AI-mediated feedback and help inform the equitable incorporation of AI into language learning contexts.

Second, the dissociation between perception and behavior, where students revised more when they believed feedback came from AI despite rating it less favorably, carries practical implications for feedback design. Given that teacher feedback is generally preferred over AI-generated feedback, AI-generated feedback should be positioned as supplementary support rather than a replacement for teacher feedback input [20, 58]. More specifically, the finding that students in the AI-label group made significantly more replacements (Cohen’s *d* = 0.70), as well as consistently more deletions and additions, suggests that AI-generated feedback may be particularly effective as a preparatory tool. In contexts where teachers face heavy feedback workloads, students could initially use AI to revise and improve their drafts before submitting them for teacher evaluation. This greater behavioral engagement with AI-attributed feedback, despite its lower perceived quality, further suggests that students may feel more comfortable experimenting with revisions when the evaluative pressure associated with teacher judgment is reduced.Teachers might also invite students to compose rebuttal letters in response to AI-generated comments to encourage deeper engagement in L2 writing. When clearly framed as a supportive and preparatory tool, AI-generated feedback may enhance students’ confidence and willingness to revise.

Third, the finding that students held less positive perceptions of AI-generated feedback across multiple dimensions (accuracy, elaboration, utility, and interest), highlights the need to address learners’ subjective attitudes toward AI tools. Effective feedback design must therefore take into account learners’ subjective attitudes toward AI tools. Negative or skeptical perceptions may discourage students from engaging with AI-generated feedback, especially when the feedback volume is high and cognitively overwhelming. To counter such biases, teachers can help learners develop an informed understanding of the affordances and limitations of AI systems, fostering strategic and critical use of AI-generated feedback. Furthermore, given that the AI-label group reported slightly higher intention to receive additional feedback despite rating the feedback less favorably, educators should consider how to harness this willingness to engage while addressing underlying perceptual biases. Additionally, as excessive or overly critical comments may activate learners’ self-defensive responses and heighten attention to negative aspects of performance [5, 22], the quantity and tone of AI-generated feedback should be carefully mediated to create an emotionally supportive learning climate.

## Conclusion

This experimental study provides empirical evidence of subjective bias in students’ responses to AI-generated feedback among a group of international students learning Chinese as a second language. The findings reveal that students tend to perceive identical feedback more favorably when it is attributed to an experienced teacher. Conversely, when informed that the feedback originates from an intelligent AI system, they are more likely to act upon it, making a greater number of revisions. These results underscore the social-affective dimension of feedback reception and offer new insights into why students may demonstrate heightened engagement when responding to AI-generated feedback. Overall, the study advances understanding of how source cues shape students’ feedback perceptions and subsequent revisions. The findings of the study point to the need for exploration into the mechanisms behind feedback perceptions, uptake, and engagement in human-AI-generated feedback contexts. Concrete implications for designing and integrating AI-generated feedback into language learning are also discussed.

This study has several limitations. First, the study was carried out in a controlled laboratory environment, which may constrain the applicability of its findings to authentic, real-world educational settings. In authentic classroom settings, students’ engagement with feedback can be shaped by multiple factors, including the importance of the writing task, the nature of the teacher–student relationship, and students’ perceptions of the potential costs and benefits of seeking feedback. Whether subjective bias exists in authentic settings and to what extent such bias influences textual revisions therefore merit further investigation in replication studies. Second, the composition of the sample may also limit the generalizability of the findings. Although the participants represented multiple nationalities, approximately 75% were descendants of overseas Chinese. This demographic profile may influence learners’ cultural attitudes toward authority and teacher evaluation, which in turn could shape their perceptions of feedback sources. Future research involving more culturally diverse samples would help determine whether the observed patterns hold across different educational and cultural contexts. Third, the study focused solely on participants’ revision frequency without examining the quality of the revisions made. The interaction between biased perceptions, feedback quantity, and revision quality in shaping feedback engagement also merits further scholarly attention. Fourth, the questionnaire was pilot-tested by only two participants, which may have compromised the reliability and validity of the self-report measures. Future research should therefore test the questionnaire with a larger sample to ensure its suitability for the target population and intended constructs.

## Supplementary Information

Supplementary Material 1.

## Data Availability

The datasets analyzed during the current study are available from the corresponding author on reasonable request.

## Appendix A

### The essay

吸烟对个人健康和公共利益的影响.

吸烟有害健康这个道埋众所同知。随着人们文化素质和生活水平的提高, 越来越多公共场所实行对吸烟者的惩罚措施也是完全合乎情理的。因为吸烟不仅有害个人健康, 也同时给他人和市容环境带来多种伤害。

很多青少年寻求酷或刺激, 还未了解吸烟会造成的种种后果时便染上了这个坏毛病。给自己的人生带来众多不益。市政府限制吸烟场所的规定若能阻止哪怕是一个青年陷入自我伤害的陡境, 就是应该支持的。

再来说吸烟者对环境的破害。首先, 烟物本身就是污染, 除此之外, 乱丢烟头也是普遍的现象。所以, 为了保持市容美丽整齐, 限制吸烟场所也是有必要的。更何况很多场所设有易燃易爆物品。

市政府对吸烟加以限制是出于很坚定的立场: 为了保护市容和居民。所以这一新规定并没有什么不妥。希望广大群众会因此更加深刻的体会并且认识吸烟的害处。

## Appendix B

### The feedback points

总体评价:

1. 总体内容和结构: 文章内容较为充实, 结构基本完整, 但部分内容的逻辑性和条理性可以进一步加强。例如, 可以更清晰地组织青少年吸烟问题和环境问题之间的关系。
2. 语法：文章语句基本通顺，但有一些语法错误和用词不当，如“道埋众所同知”应为“道理众所周知”，“破害”应为“破坏”，“陡境”应为“困境”。
3. 汉字和词汇：文章中的词汇使用有一定的丰富性，但有些词语使用不够准确或恰当，如“酷”应为“酷感”，“不益”应为“不利”。
4. 内容：文章内容较为充足，能够覆盖吸烟对健康和环境的影响，但可以进一步提供更多具体的例子或数据来支持观点。
5. 任务要求：文章较好地回答了关于吸烟危害的问题，但可以更全面地涵盖任务要求，例如，可以讨论不同公共场所对吸烟的不同限制措施。
6. 可理解性：文本基本上是可以理解的，但有些句子不够清晰，需要读者再次阅读以理解作者的意图。
7. 衔接和连贯：文本在某些部分的衔接和连贯性有待提高，可以通过使用更多的连接词和过渡句来增强文章的流畅性。

修改建议:

1. 修正语法错误和用词不当。
2. 使用更准确的词汇和表达。
3. 加强论点之间的逻辑性和条理性。
4. 使用更多的连接词和过渡句来提高文章的连贯性。
5. 提供更多具体的例子或数据来支持观点。

## Appendix C

### Feedback perception questionnaire

#### Coverage

1. 反馈点评了作文的词汇和语法。
  The feedback considers the words and grammar of the essay.
2. 反馈点评了作文的主题和内容。
  The feedback commented on the theme and content of the essay.
3. 反馈指出了作文存在的问题。
  The feedback pointed out the problems of the essay.
4. 反馈肯定了作文的优点。
  The feedback affirms the strengths of the essay.
5. 整体来说, 反馈比较全面。
  Overall, the feedback is comprehensive.

#### Accuracy

1. 反馈对词汇使用的评价是准确的。
  The feedback on the use of words is accurate.
2. 反馈对语法方面的评价是准确的。
  The feedback on the grammar is accurate.
3. 反馈对作文的思想和内容的评价是准确的。
  The feedback on the content of the essay is accurate.

#### Elaboration

1. 反馈指出了错误的性质。
  The feedback points out the nature of errors.
2. 反馈解释了错误的原因。
  The feedback explains why it is an error.
3. 反馈提供了修改的方向。
  The feedback provides a direction for revision.
4. 总的来说, 无论是正面反馈还是负面反馈, 都有理有据。
  Overall, the feedback, whether positive or negative, is based on grounds.

#### Interest value

1. 对这篇作文的反馈有意思。
  The feedback on the essay is interesting.
2. 读这篇作文的反馈评价很有趣。
  It is fun to read the feedback on this essay.
3. 从反馈中我学到了新知识。
  I learned new things from this feedback.
4. 如果是我写的这篇作文, 读完反馈, 我会有动力写得更好。
  If I wrote the essay, I would be more motivated to write better after reading the feedback.

#### Utility value

1. 这篇反馈有助于作文修改。
  The feedback is useful for essay revision.
2. 这篇反馈有助于作者评估自己的水平。
  The feedback is useful to help the writer evaluate his proficiency
3. 这篇反馈有助于作者提升自己的写作水平。
  The feedback can help the writer enhance his writing proficiency

#### Cost

1. 阅读这篇反馈花费很多时间和精力。
  Reading the feedback costs time and effort.
2. 这篇反馈指出了很多错误, 让人觉得难堪。
  The feedback pointed out many errors, which makes people feel embarrassed.
3. 这篇反馈让人感觉有压力。
  The feedback causes pressure.
4. 这篇反馈让人有挫败感, 会影响学习动力。
  The feedback makes people upset and demotivated.

#### Intention to receive feedback

1. 总体而言, 我觉得这篇评语很好.
  Overall, I think the feedback is very good.
2. 在以后的写作中, 我也希望能收到这样好的反馈。
  I hope to receive such feedback on my future essays.
3. 如果以后都能收到这样好的反馈, 我会很满意。
  I will be very satisfied if I can receive such good feedback in the future.
