# Supplementary material for: Students’ psychological biases towards teacher and AI-generated feedback: an experimental study
Source: BMC Psychol. 2026 Apr 22;14:818. doi: 10.1186/s40359-026-04568-5 (PMC13235111; doi:10.1186/s40359-026-04568-5)
Supplement: Supplementary file 1 — Supplementary Material 1. [file 40359_2026_4568_MOESM1_ESM.docx]

**Feedback Perception Questionnaire**

Below is a series of statements about the quality of the feedback comments you have just read. Please indicate the degree to which you agree with the statements. 1 indicates “very disagree” and 5 indicates “very agree”. This is not a test. So, there is no right or wrong answer. Please express your true thoughts.

| **Coverage** | | | | | |
| --- | --- | --- | --- | --- | --- |
| 1.The feedback considers the words and grammar of the essay. | 1 | 2 | 3 | 4 | 5 |
| 2.The feedback commented on the theme and content of the essay. | 1 | 2 | 3 | 4 | 5 |
| 3.The feedback pointed out the problems of the essay. | 1 | 2 | 3 | 4 | 5 |
| 4.The feedback affirms the strengths of the essay. | 1 | 2 | 3 | 4 | 5 |
| 5.Overall, the feedback is comprehensive. | 1 | 2 | 3 | 4 | 5 |
| **Accuracy** | | | | | |
| 1.The feedback on the use of words is accurate. | 1 | 2 | 3 | 4 | 5 |
| 2.The feedback on the grammar is accurate. | 1 | 2 | 3 | 4 | 5 |
| 3.The feedback on the content of the essay is accurate. | 1 | 2 | 3 | 4 | 5 |
| **Elaboration** | | | | | |
| 1.The feedback points out the nature of errors. | 1 | 2 | 3 | 4 | 5 |
| 2.The feedback explains why it is an error. | 1 | 2 | 3 | 4 | 5 |
| 3.The feedback provides a direction for revision. | 1 | 2 | 3 | 4 | 5 |
| 4.Overall, the feedback, whether positive or negative, is based on grounds. | 1 | 2 | 3 | 4 | 5 |
| **Interest** | | | | | |
| 1.The feedback on the essay is interesting. | 1 | 2 | 3 | 4 | 5 |
| 2.It is fun to read the feedback on this essay. | 1 | 2 | 3 | 4 | 5 |
| 3.I learned new things from this feedback. | 1 | 2 | 3 | 4 | 5 |
| 4.If I wrote the essay, I would be more motivated to write better after reading the feedback. | 1 | 2 | 3 | 4 | 5 |
| **Usefulness** | | | | | |
| 1.The feedback is useful for essay revision. | 1 | 2 | 3 | 4 | 5 |
| 2.The feedback is useful to help the writer evaluate his proficiency. | 1 | 2 | 3 | 4 | 5 |
| 3.The feedback can help the writer enhance his writing proficiency. | 1 | 2 | 3 | 4 | 5 |
| **Cost** | | | | | |
| 1.Reading the feedback costs time and effort. | 1 | 2 | 3 | 4 | 5 |
| 2.The feedback pointed out many errors, which makes people feel embarrassed. | 1 | 2 | 3 | 4 | 5 |
| 3.The feedback causes pressure. | 1 | 2 | 3 | 4 | 5 |
| 4.The feedback makes people upset and demotivated. | 1 | 2 | 3 | 4 | 5 |
| **General impression** | | | | | |
| 1.Overall, I think the feedback is very good. | 1 | 2 | 3 | 4 | 5 |
| 2.I hope to receive such feedback on my future essays. | 1 | 2 | 3 | 4 | 5 |
| 3.I will be very satisfied if I can receive such good feedback in the future. | 1 | 2 | 3 | 4 | 5 |

**Personal Basic Information**

1. Gender: __________

2. Age: __________

3. Nationality: __________

4. Grade: Undergraduate ( ) Grade ______or Postgraduate ( ) Grade_______

5. Major: ________________

6. Which languages can you speak (proficiently)? ______________________________

7. Have you taken the HSK test? (If “Yes”, please answer the next question) A. Yes B. No

8. What level and score did you get? ______________________________

9. Are you of Chinese descent? A. Yes B. No
